# Analysis of the Anisotropy of Sound Propagation Velocity in Thin Wooden Plates Using Lamb Waves

**DOI:** 10.3390/polym16060753

**Published:** 2024-03-09

**Authors:** Dagmar Faktorová, Mariana Domnica Stanciu, Michal Krbata, Adriana Savin, Marcel Kohutiar, Milan Chlada, Silviu Marian Năstac

**Affiliations:** 1 Faculty of Special Technology, Alexander Dubcek University of Trenčín, 911 06 Trenčín, Slovakia; dagmar.faktorova@tnuni.sk (D.F.); michal.krbata@tnuni.sk (M.K.); marcel.kohutiar@tnuni.sk (M.K.); 2 Faculty of Mechanical Engineering, Transilvania University of Brașov, 29 Eroilor Blvd., 500036 Brașov, Romania; silviu.nastac@ugal.ro; 3 National Institute of Research and Development for Technical Physics, 47 D. Mangeron Blvd., 700050 Iasi, Romania; 4 Institute of Thermomechanics of the CAS, v. v. i. Dolejškova 1402/5, 18200 Prague, Czech Republic; chlada@it.cas.cz; 5 Faculty of Engineering and Agronomy, Braila, 810017, “Dunarea de Jos” University of Galati, 810017 Galati, Romania

**Keywords:** varnish, anisotropy, Lamb waves, coating treatments, spruce, maple

## Abstract

The objective of the study was to analyze the influence of coating treatments on sound propagation speeds in thin boards, along the longitudinal and radial directions of resonance wood. The samples studied were thin boards made of spruce and maple wood with dimensions of 240 mm × 80 mm × 4 mm (length × width × thickness) subjected to different coating treatments (oil-based varnish and alcohol varnish) as well as unvarnished samples, exposed to radiation UV, and specimens treated in the saline fog. The test method consisted of evaluating the propagation speeds of Lamb waves applied to thin plates, according to a semicircular test model, so that the results highlighted both the acoustic response in the longitudinal and radial directions as well as the variation in the anisotropy of the samples with the change in the sound propagation direction relative to wood fibers. Based on the statistical analysis, sound propagation speed profiles were obtained in each of the 38 directions analyzed for all wood samples. The results highlighted that the oil-based varnish led to a decrease in the speed of propagation in the radial direction, compared to the alcoholic varnish, whose major effect was in the longitudinal direction, on the spruce wood. On maple wood, increasing the number of varnish layers, regardless of the type of varnish, led to a decrease in the anisotropy ratio between the longitudinal and radial directions.

## 1. Introduction

Wooden material, due to its remarkable properties, traditionally presents wide applicability. Wood, through its cellular structure, shape and way of organizing the wood cells in the three main directions, and content of lignin, cellulose, and hemicelluloses, confers optimal physical, mechanical, and acoustic properties for musical instruments with strings and a resonator body. The physical properties (density) and mechanical properties of wood (elastic constants and damping) influence the acoustic properties of wood (speed of sound, characteristic impedance, and sound radiation) because sound is produced by the vibrations of the material [1,2,3]. According to [2], the parameters that reflect the vibrational and acoustic quality of wood are the relative acoustic conversion efficiency as the ratio of acoustic energy radiated to the vibration energy, the anisotropy of wood calculated as the ratio between dynamic Young’s modulus and dynamic shear modulus. Thus, a high specific dynamic modulus in the longitudinal direction, a high anisotropy factor, and a low damping are indicators of the tone quality of the soundboard. From the point of view of the fiber orientation compared to the cutting directions of the wooden boards, [3] considers that the grain angle influences the anisotropy of viscoelastic vibrational properties, just as [2,4] correlates the acoustic qualities of wood with the effect of the angle of the microfibril on the damping and specific dynamic Young’s modulus.

On the other hand, the accessibility of this resource since ancient times and its easy workability have consecrated wood as a lignocellulose composite material for numerous musical instruments [1].

The reaction of wood in the interaction with sound waves depends on the transmitted sound energy, as well as the nature and condition of the wood: the macro- and microscopic structure of the wood–cell, membrane construction, fiber size, cohesion, humidity, elastic properties, etc. Spruce and maple wood (resonance wood) are wood with a very fine structure and physical properties suitable for the construction of stringed musical instruments [5,6]. 

According to [7,8], who identified the origin of the resonance wood of the musical instruments belonging to the Cherubini Conservatory Collection in Florence, the most important areas for the production of soundboards were the Eastern Alps (with regions from Italy and Slovenia), Bavarian forests, and the Carpathian Mountains (Poland, Romania, and southern Russia). The resonance wood from the central–northern area of the Eastern Carpathians presents special physical and acoustic properties that have been studied, with them being correlated with the anatomical quality classes used by luthiers [8,9,10,11]. The choice of raw material by luthiers is based on rigorous physical and acoustic criteria, with the resonance wood thus being classified in correlation with the quality and price of the musical instrument. The properties of the materials used in the construction of stringed musical instruments show great variability, and that is why the first stage in the manufacture of musical instruments is the choice of wood [12,13,14]. The basic characteristic that distinguishes resonance wood from the ordinary type is the very homogeneous anatomical structure, obtained from the small and uniform width of the annual rings as well as the small proportion of late wood. The microscopic structure of spruce wood consists of vessels, resin canals, and cellular composition. The length of the tracheids shows a significant increase when moving away from the pith and parcel. The vessels in the ring assembly are 1.1–6.0 mm long. Tracheid length decreases with latitude. The resin canals are visible only with a magnifying glass and are rare (25–35 pcs/cm^2^). The cellular composition consists of 95% tracheids, 3.6% resiniferous formations, 1.4% radial parenchyma, and a proportion of wood voids of 71% [15,16,17].

From an anatomical point of view, maple wood is made up of vessels (trachea), wood parenchyma, radial parenchyma, and fibers. The vessels are small and rare, and the fibers occupy about 75% of the volume of the wood substance. The porosity (proportion of voids) is 60% of the apparent volume. Maple wood is part of the category of hardwoods with scattered pores, with these being small, visible with a magnifying glass, and quite rare. They are, therefore, unevenly scattered among the early and late wood and their size and thickness decrease towards the outer limit of the annual ring.

Producers choose wood according to its cost and mechanical, artistic, and mechanical processing criteria without, however, having the guarantee of the performance of the final product, even in the conditions of the best acoustic performance of the wood [3,8]. The wood used in the construction of musical instruments is covered with lacquer films that have the role of protecting the musical instrument from wear and fluctuations in ambient humidity. Depending on the type of varnish (the solvent), the thickness of the film, the degree of penetration into the wood, the physical and mechanical properties of the newly formed system (wood–wood/varnish interface and the varnish film) show elastic, acoustic, and dynamic properties different from those of untreated wood [2,3,4].

Lately, there has been increased interest in scientific research on these materials by applying direct and indirect methods and the appropriate use of measuring instruments [16]. Non-destructive techniques (NDT/E) involve the identification of properties without measuring them and preserving the structural integrity of the wood [11,12,13]. Simulation methods using the finite element method (FEM) can be used for the evaluation techniques of material properties even when they have complex behavior [11,12,13]. Ultrasonic (US) and ultrasonic resonance (RUS) methods as part of NDT/E methods successfully achieve the results obtained by standard static methods [14,15,16,17]. 

The results obtained in determining the propagation speeds of US in wood depend on the orientation of the structure in relation to the sound source (longitudinal—L, radial—R, and tangential—T) [18,19,20,21], according to the axes that identify its spatial directions. The propagation speed of US through wood is a fundamental parameter in the characterization of resonant wood, with it being dependent on the density of the wood as well as the modulus of elasticity [22,23]. From a mechanical point of view, to understand the elastic behavior of resonant wood, it is necessary to determine the propagation speeds in the three directions. The method based on US propagation as a non-destructive method involves nine propagation speeds in accordance with the directions of mechanical oscillation [22,23,24]. According to [20,21], the structural anisotropy of wood can be determined using different methods: either by calculating the ratios between the longitudinal and transverse wave velocities in the three main directions of symmetry or based on the ratios between shear velocities, or through the estimation of wood anisotropy deals with velocity invariants. The analysis of the anisotropy of wooden plates based on the propagation of Lamb waves provides useful information not only about the propagation velocity of horizontal shear waves but also the dispersion phenomena that depend on the geometry of the structure, the dependence between the wave number, and the frequency of the particular mode that can be obtained by a numerical solution of the Rayleigh–Lamb frequency equation [25,26,27]. According to [18,28,29], for bulk waves, it is already well known that the anisotropy implies a difference between the group velocity and the phase velocity. It is shown here that this phenomenon also occurs for Lamb waves, with it affecting both the speed and direction of propagation.

This paper aims to emphasize the effect of the acoustic anisotropy of wood as a result of the preferential orientation of the anatomical elements and the different surface treatments applied to spruce and maple wood. The novelty of the study consists of the analysis of the acoustic anisotropy of wood subjected to different surface treatments (artificial aging, salt spray, and different types of finishes). 

## 2. Materials and Methods

### 2.1. Materials

**Figure 1:**
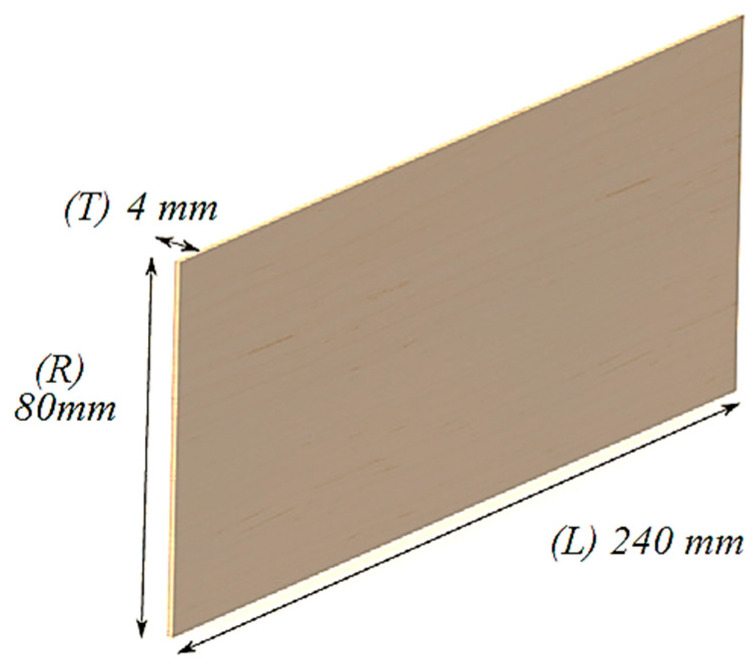
The geometry of the samples.

Spruce (*Picea abies Karst* L.) and maple (*Acer pseudoplatanus* L.) wood specimens were included in the tests, with dimensions 80 × 4 × 240 mm^3^ corresponding to the radial × tangential × longitudinal directions, which were tested (according to ISO13061-1:2014/Amd1:2017 [30]) under laboratory conditions (20 °C and relative humidity (RH) of 65%). In Figure 1, the geometry and physical aspects of the samples are presented. From the point of view of the anatomical structure of the wood, two quality classes were analyzed: class A representing wood with a fine anatomical structure, with narrow and regular annual rings (for spruce wood) and wavy fibers (for maple wood), and class D representing wood with a coarser anatomical structure, with annual rings wider and irregular in width (for spruce wood) without wavy fibers (for maple wood) [10,23].

The samples were subjected to different surface treatments to check to what extent these treatments influence the degree of anisotropy of the wood. Table 1 shows the characteristics of the investigated samples. The moisture content was monitored using a Merlin HM8-WS1 (Tumeltsham, Austria) moisture meter and the mass was measured with a Kern & Sohn Weighing Balance, EWJ 600-2SM, Balingen, Germany.

The samples were obtained by cutting radially longitudinally from the boards, which, after natural drying, were conditioned in a drying chamber up to a moisture content of 6–8%. Both nowadays and in the past, stringed musical instruments were covered with varnish to protect them from variations in atmospheric humidity and dirt [31,32,33]. Current studies with modern methods of determining the chemical fingerprint of the varnish layers on old violins have highlighted the fact that luthiers used recipes based on alcohol-solvent resins, with oils, or the wood was treated with salts like borax and the sulfates of Zn, Cu, Cr, Na, and Fe [32,34]. Based on these considerations, currently, the most well-known types of varnishes used for maestro- and professional-level musical instruments are those with an oil-based solvent and those with an alcoholic solvent (spirit) [32,33,34,35]. Not only is the chemical composition of the surface treatment important but also the thickness of the varnish film and the varnish–wood interface, aspects highlighted in studies [36,37,38]. The type of varnish and its thickness correlated with the number of layers applied in the finishing process influence the tonality of the musical instrument as a result of not only the difference in stiffness between the wood and the varnish film but also as an effect of the increase in the mass of the layered material (wood–varnish) [31,32,38]. Thus, in the presented study, spruce and maple wood samples covered with a number of layers of lacquer were analyzed according to the procedures applied at a violin factory (5, 10, and 15 layers). Therefore, some of the samples were kept as control samples, others were exposed to artificial aging with UV radiation, another set of samples were varnished with oil-based varnish and spirit varnish with a different number of layers (5, 10, 15), and another set of samples were exposed to the salt fog, as can be seen in Table 1. 

According to [36,38], to compare the mass changes of the spruce and maple boards before and after surface treatments (VS varnish system, UV exposure, and salt fog exposure), the changes were evaluated as area mass loading (AML) calculated based on mathematical relation (1):

(1) $$AML=\frac{m_{t}-m_{w}}{b*l},$$

where AML is area mass loading induced by treatment (g·m^−2^); $m_{t}$—the final mass of the treated wood; $m_{w}$—the initial mass of the wooden sample; *b*—the width of the plate; and *l*—length of the plate [36,38].

### 2.2. Methods

#### 2.2.1. Experimental Set-Up

Basically, ultrasound (US) non-destructive evaluation consists of applying physical elastic waves to the sample tested and analyzing the interaction between the material samples and the field [19,39,40,41].

**Figure 2:**
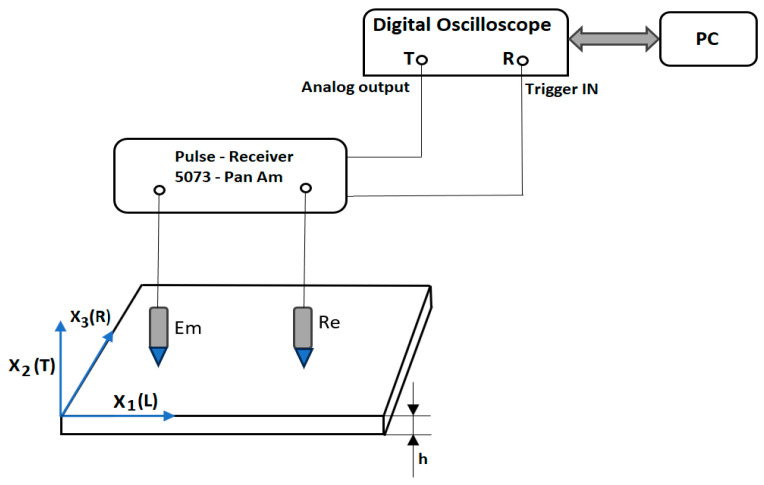
Experimental set-up.

A problem associated with the propagation of Lamb waves is the coexistence of several modes of oscillation in the plate, which leads to difficulties in interpreting the results. The higher-order modes of oscillation start to propagate when the thickness of the plate becomes comparable to the wavelength of the longitudinal and transverse waves. Lamb waves are guided elastic plate waves that can be described as a combination of compression waves (P-waves) and shear waves (S-waves) [22,42]. For the excitation of Lamb waves in the lowest mode, transducers with Hertzian contact are used. A diagram of the system that generates and detects the Lamb waves for measurement is presented in Figure 2.

A piezo-electric transducer, of very low eigenfrequency (tens of kilohertz), is coupled to a buffer rod made of a material of elasticity module E_1_ and Poisson coefficient ν_1_ which has a spherical bumped head of radius R at one end. The buffer rod is compressed onto a plate (with elasticity module E_2_ and the Poisson coefficient ν_2_). To increase the reliability and assurance of the quality of measurement, the ultrafine force presses the US sensors at constant value. The speed of the Lamb waves generated by Hertzian contact was determined, placing the emission and the reception transducer on the sample taken into study, measuring with ±10 µm precision the distance between the centers of the transducer and measuring with 0.1µs precision the time between the generation of the Lamb wave and its reception. The two buffer rods of the US sensors used at the emission and reception are both identical, with them being made of the 7075-T6 aluminum–magnesium alloy, with a density of 2.7 × 103 kg/m^3^, Young’s modulus of 7 × 1010 N/m^2^, Poisson coefficient of 0.34, and point curvature radius of 2 mm. The US sensors are connected to a 5073PR Pulse Receiver—equipment from Panametrics NDT USA (Olympus Corporation, Waltham, MA, USA). The visualization of the signal and the measurement of the time of propagation were made using a Le Croy Wave Runner 64Xi digital oscilloscope (LeCroy Corporation, Chestnut Ridge, NY, USA), with it having a sampling frequency of 10G S/s [23,24].

**Figure 3:**
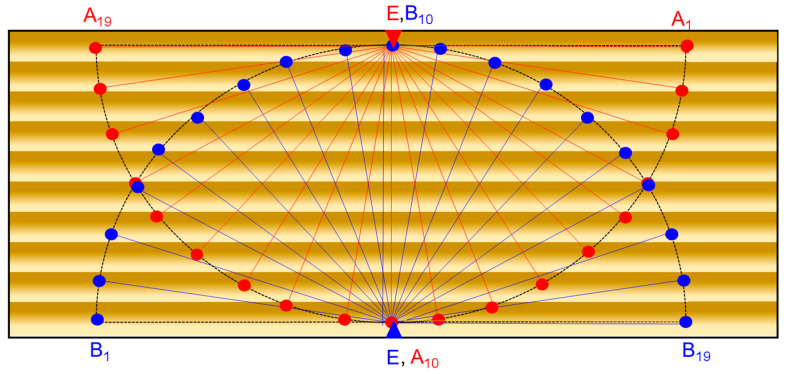
The measured points on the samples.

The signals emitted by the transmitter positioned at point E (Figure 3) were successively measured at points B1–B19 and A1–A19 as points on a semicircle, located at an interval of 10 degrees and a radius of 70 mm. The triangle symbol in points denoted E represents the position of the transmitter, and the circle symbol arranged in the form of a semicircle or quarter circle represents the successive position of the receiver. In order to avoid the dissipative behavior of the Lamb waves in the wood specimens, an equidistant distribution of the position of the transducers was chosen so that the effect of the anisotropy of the wood structure could be highlighted [18,27,28,29].

#### 2.2.2. Statistical Analysis

The data were analyzed to evaluate the anisotropy of the material samples in terms of the change in the speed of sound with respect to the direction of propagation. The distribution of resonance points on a board made of a given type of material seems to be largely influenced by the changes in the speed of sound propagation in different directions and can affect the acoustic impression of the musical instrument produced. The question remains as to the overall subjective assessment of the quality of the instrument, which is influenced by other factors but cannot be captured by the distribution of sound velocities alone. In this limited sense, the data were analyzed with a focus on selecting the most significant wood samples. Factor analysis (FA) was used as a basic tool to identify possible dependencies in the data. Based on the Q-factor analysis (QFA), several clusters of samples with similar anisotropy were extracted.

## 3. Results and Discussion

### 3.1. Grain Angle Dependence of Lamb Wave Velocity in Wooden Plates

**Figure 4:**
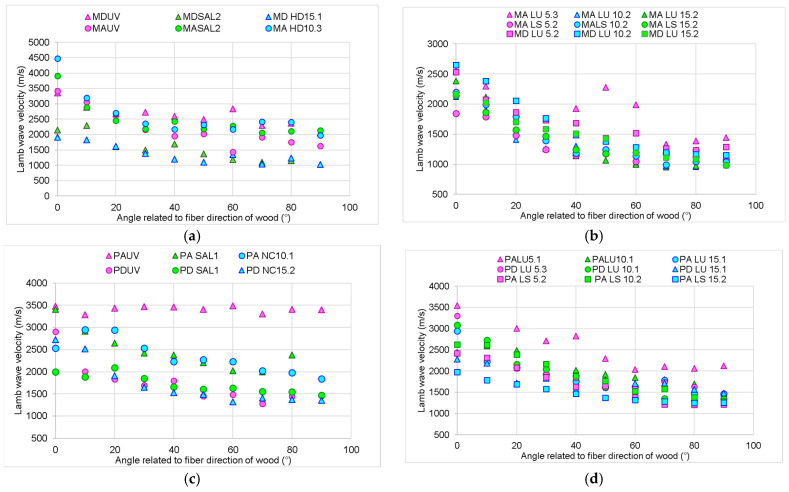
The variation in Lamb wave velocity in wooden plates with fiber angle variations: (**a**) spruce wood sample untreated, UV aged, and salt fog exposed; (**b**) spruce wood sample finished with oil-based varnish and spirit varnish; (**c**) maple wood sample untreated, UV aged, and salt fog exposed; (**d**) maple wood sample finished with oil-based varnish and spirit varnish.

Since wood is an anisotropic material both in terms of morphology and properties, the changes regarding the wave propagation capacity vary gradually with the direction of the fibers. In Figure 4, it can be seen that for all samples, the speed of Lamb waves in the radial direction ranges between 1.4 to 3 times lower than in the longitudinal direction, depending on the species, the structure of the wood, and the type of treatment applied.

**Figure 5:**
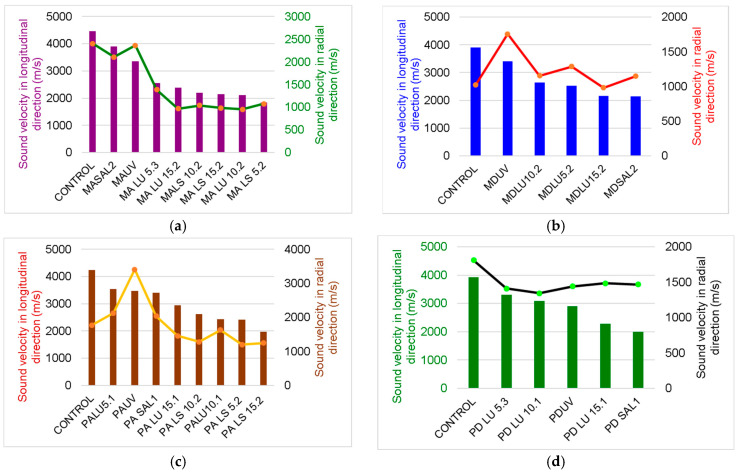
The influence of surface treatment on longitudinal and radial wave velocity in wooden plates: (**a**) spruce wood sample for maestro musical instruments; (**b**) spruce wood sample for school musical instruments; (**c**) maple wood sample for maestro musical instruments; (**d**) maple wood sample for school musical instruments.

The treatments applied to the boards influence their response to the propagation of waves in the board. Thus, artificial aging via exposure to UV radiation and slight thermal degradation for 300 h led to a decrease in both longitudinal (24%) and radial (1.7%) propagation speeds compared to the untreated wood. On the other hand, the coating with varnish films produced a wave dispersion phenomenon, with their speed decreasing in the longitudinal direction by 42–58% and the alcoholic varnish affecting the propagation of sounds the most in both spruce and maple wood (Figure 5). In the radial direction, the oil-based varnish applied on spruce wood is the one that presents the lowest values of the wave propagation speed, with it being 60% lower than the speed of the control samples. In the case of maple wood, the film of alcoholic varnish reduces the sound propagation speed the most, by 32% compared to the control sample (untreated).

**Figure 6:**
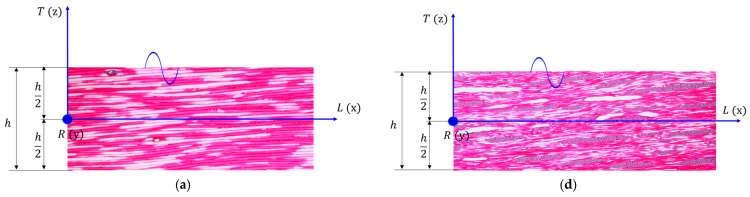

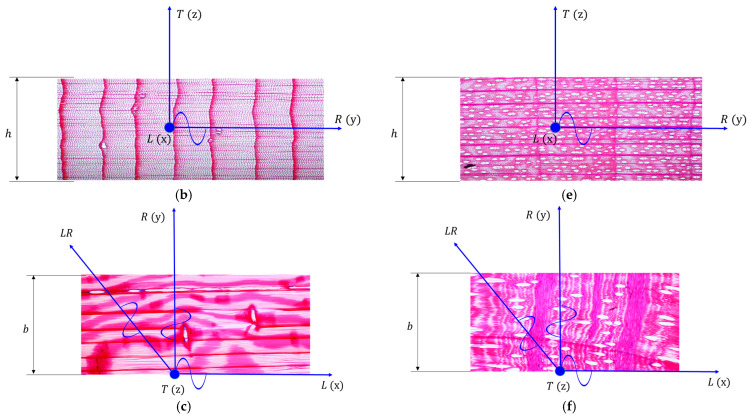
The microscopic views of the main section of wood related to the Lamb wave direction in the studied plates: (**a**) tangential section of spruce wood; (**b**) cross-section of spruce wood; (**c**) radial section of spruce wood; (**d**) tangential section of maple wood; (**e**) cross-section of maple wood; (**f**) radial section of maple wood.

Sound propagation in spruce and maple wood depends on the type, organization, and three-dimensional shape of the anatomical elements of each species. In spruce wood (*Picea abies Karst* L.) in the longitudinal direction, tracheids constitute 90% of the wood volume and are approximately 100 times longer than they are wide, with them overlapping with adjacent cells both in the upper and lower parts by 20–30% of their length. This construction ensures the rapid propagation of waves in softwood [42,43,44,45]. In Figure 6a,c, the microscopic view in the tangential direction of spruce wood in relation to the measurement principle can be noted. In the radial direction, the transmitted waves meet the tracheids whose walls show thicknesses that increase from earlywood to latewood, so it can be considered a layered material (with each annual ring being a layer) (Figure 6b).

In the radial direction, spruce wood contains radial parenchyma cells that have a geometry like rectangular prisms with dimensions of 15 µm high by 10 µm wide and 150–250 µm long in the radial or horizontal direction [8,21]. In the case of maple wood (*Acer pseudoplatanus* L.), the anatomical architecture is more complex than that of spruce wood, with it being made up of fibers, vessels, and axial parenchyma cells, with different shapes, organization, and quantity [42,43]. The vessels are small and rare, and the fibers occupy about 75% of the volume of the woody substance, the proportion of voids is 60% of the apparent volume, and from the point of view of organization, maple wood is part of the hardwoods with diffused, small pores, visible with a magnifying glass and quite rare, with their size and thickness decreasing towards the outer limit of the annual ring (Figure 6d–f) [44,45]. The vessels have average lengths between 350–800 μm, with small diameters, <50 μm. As a result, the propagation of sounds in maple wood differs from that of spruce wood; the waves propagate through a structure with a different organization and construction.

**Figure 7:**
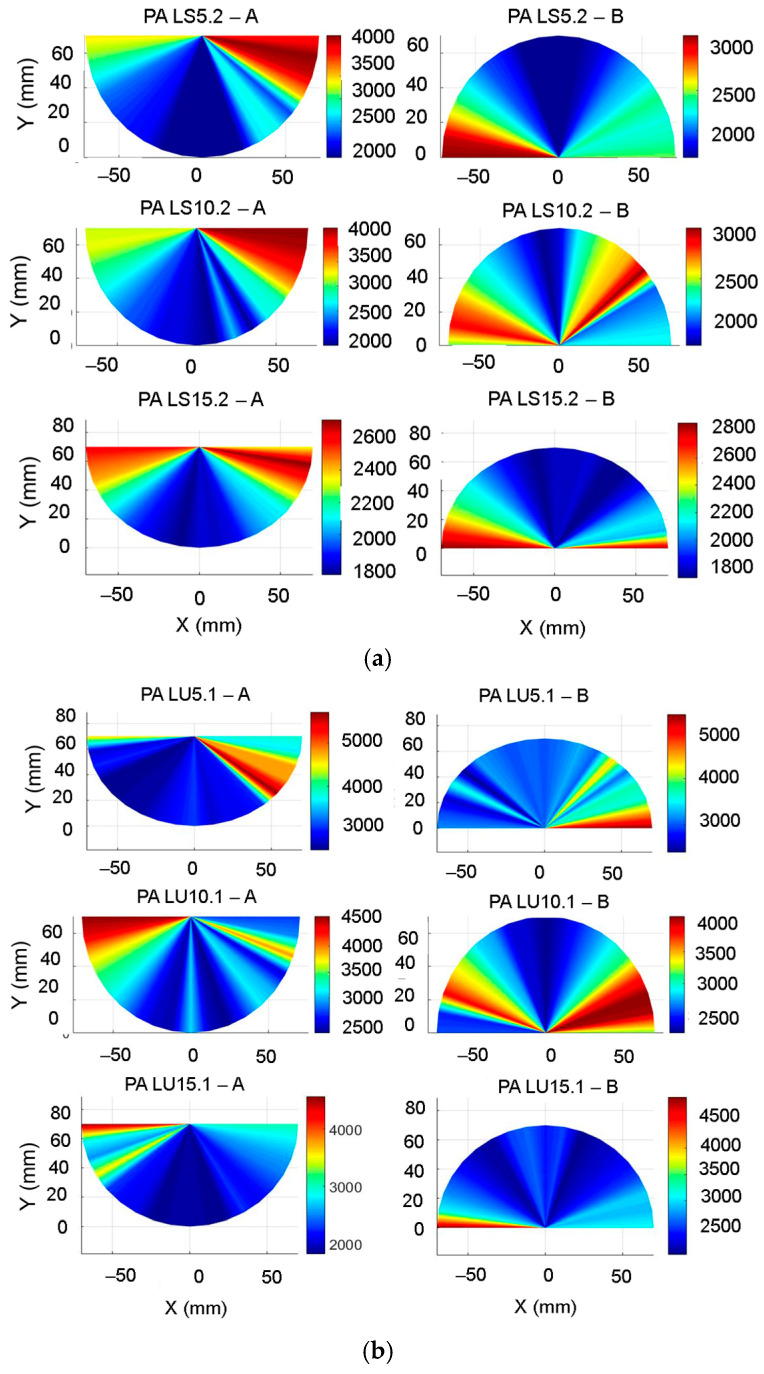

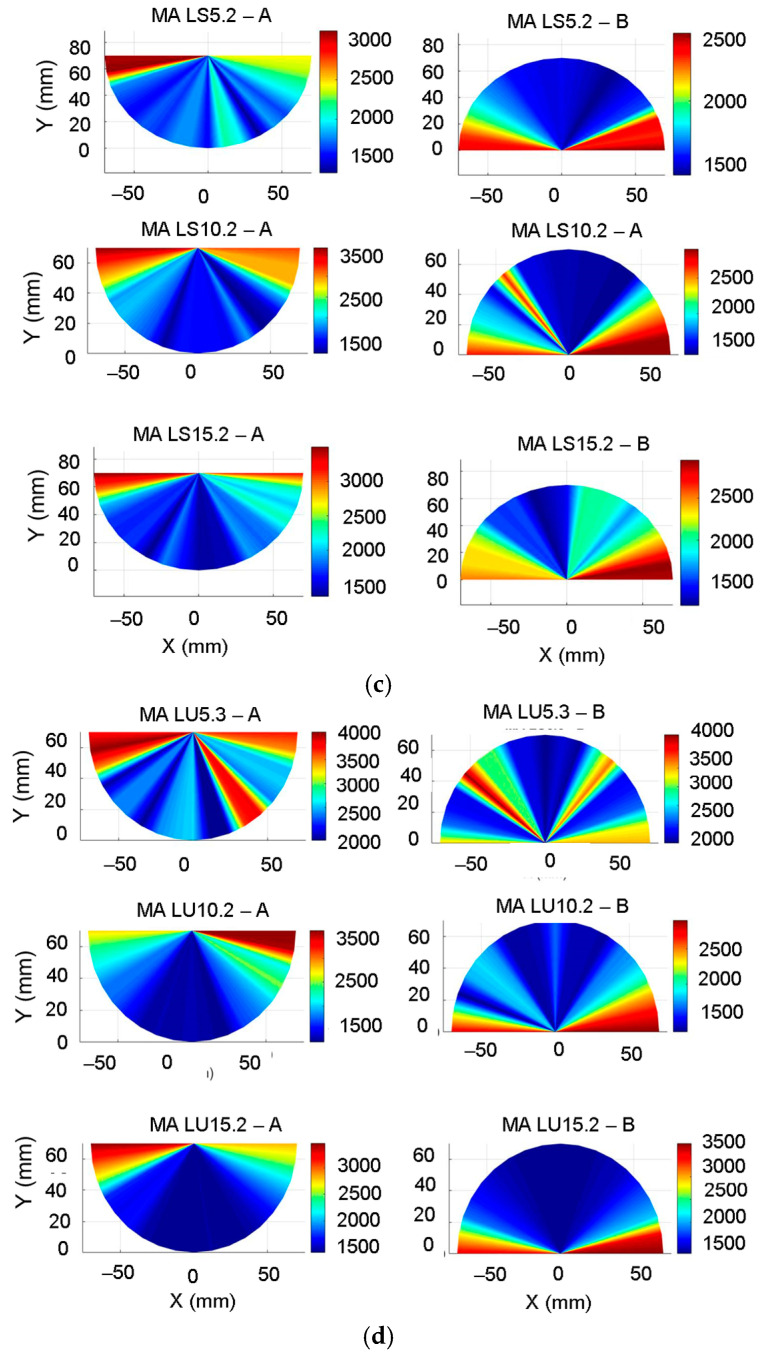
The variation in Lamb wave speeds with the rotation of the receiver position: (**a**) maple samples covered with spirit varnish; (**b**) maple samples covered with oil-based varnish and spirit varnish; (**c**) spruce samples covered with spirit varnish; (**d**) spruce wood sample finished with oil-based varnish.

Figure 7 highlights the variation in Lamb wave speeds measured on a circular trajectory applied to the wooden board. The angles of 0 and 180 degrees correspond to the longitudinal axis of the wood, and the angle of 90 degrees corresponds to the radial axis. Thus, it can be observed that between the two directions, L and R, the structural anisotropy of the wood is at its maximum, results highlighted in other studies [18,19,20,21].

Similar results were reported by [46], who analyzed spruce and sycamore wood samples from three types of quality classes: the highest, the moderate quality class, and the control samples, obtaining the following values for the investigated elastic and acoustic parameters: for high-quality spruce wood samples versus a control wood sample, the sound velocity in the longitudinal $c_{L}$ and radial $c_{R}$ directions of $c_{L}=5103\pm 280\;m/s$ and $c_{R}=1365\pm 201\;m/s$ versus the control wood sample $c_{L}=5388\pm 134\;m/s$ and $c_{R}=1124\pm 93\;m/s$. For sycamore, ref. [46] obtained, $c_{L}=3894\pm 310\;m/s$ and $c_{R}=1662\pm 97\;m/s$. Estimations of the elastic and acoustic properties were made using methods based on ultrasound [21], reported for resonance spruce with the following longitudinal velocities $c_{L}=5600\;m/s$; tangential velocity $c_{T}=1600\;m/s$; and radial velocity $c_{R}=2000\;m/s$, and for shear velocity, $c_{LT}=1425\;m/s$; $c_{RT}=298\;m/s$; and $c_{LR}=1374\;m/s$ and for common spruce, $c_{L}=5353\;m/s$; $c_{T}=1146\;m/s$; and $c_{R}=1580\;m/s$; and for shear velocity, $c_{LT}=1230\;m/s$; $c_{RT}=477\;m/s$; and $c_{LR}=1322\;m/s$. For curly maple, the values determined using ultrasonic method are: $c_{L}=4350\;m/s$; $c_{T}=1914\;m/s$; and $c_{R}=2590\;m/s$, and for shear velocity $c_{LT}=1468\;m/s$; $c_{RT}=812\;m/s$; and $c_{LR}=1744\;m/s$, since for common Sycamore, the values are $c_{L}=4695\;m/s$; $c_{T}=1878\;m/s$; and $c_{R}=2148\;m/s$, and for shear velocity, $c_{LT}=1148\;m/s$; $c_{RT}=630\;m/s$; and $c_{LR}=1354\;m/s.$ Ref. [47] noticed that for the ratio of the sounds velocities in wood in the two longitudinal and radial directions, within the same wood species, for the spruce samples, the ratio CL:CR was 3.257, and for the samples of maple wood, the ratio was 2.17.

### 3.2. Statistical Results

**Figure 8:**
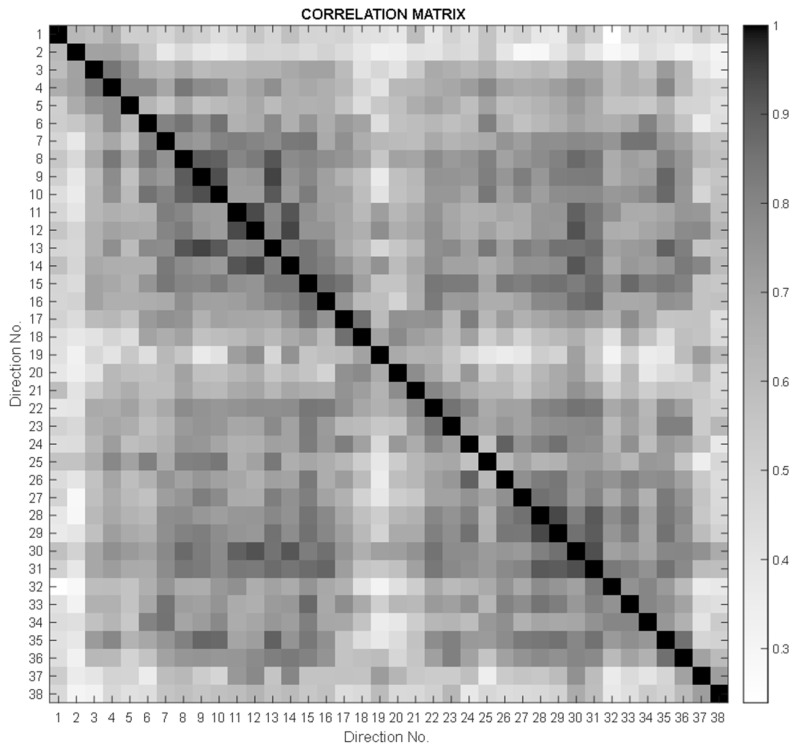
“Butterfly” distribution of correlation values.

In order to verify the continuity of the measurement points, a correlation analysis of the values collected for all material samples was performed, considering the values measured on the two semicircles A and B as forming a fictitious circle. In the correlation matrix in Figure 8, a “butterfly” distribution of correlation values can be observed, with a diagonal symmetry that corresponds to the orientation symmetry of the wood fibers.

**Figure 9:**
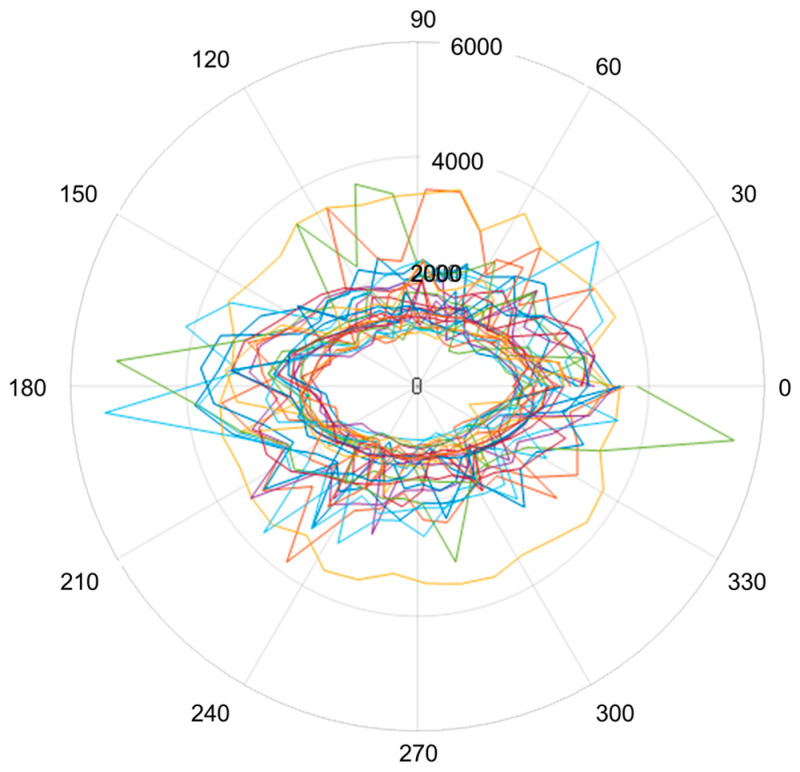
Polar graphs of sound velocities for each sample.

The anisotropy of the sound propagation speeds in the wood samples is highlighted in the polar graph of the speeds in each of the 38 directions for all of the wood samples (Figure 9).

**Figure 10:**
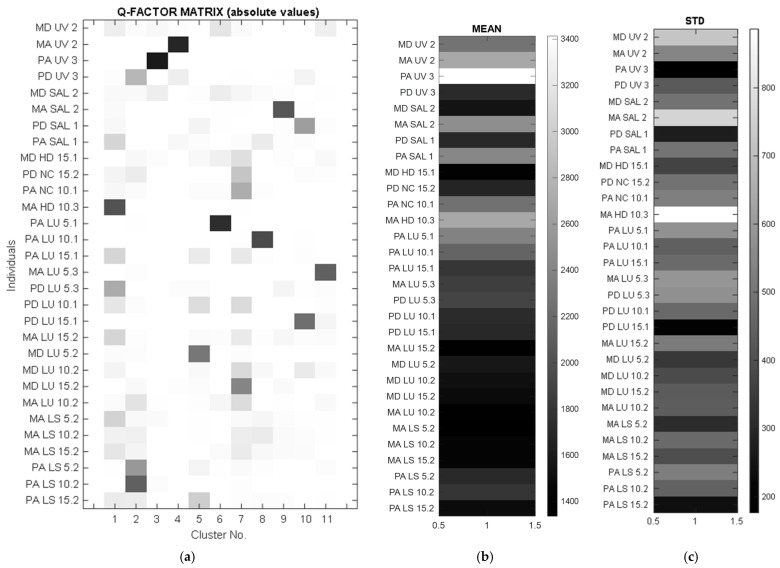
Comparative analysis of statistical data: (**a**) Q-FA clustering scheme; (**b**) the square root of the mean square values; (**c**) STD values for each sample.

This summary shows the variability of the measured values with respect to the different directional dependencies, as well as the relatively large variation in the maximum speed measured in individual samples. Using the Q-variant of FA (based on a transposed data matrix and resulting in a clustering scheme, see the left part of Figure 8), it was possible to point to certain samples that had a particular pattern of anisotropy. Based on the Q-factor analysis (QFA), several groups of samples with similar anisotropy were extracted, depending on the wood species, the applied treatment, or the anatomical characteristics. In some of these groups, only a specific sample belongs to a cluster and therefore can be declared exceptional in its own way. These selected samples are due to the type of wood and different treatment methods. Using QFA, the measured parameters are ordered into a so-called individual profile, which helps to identify similar characteristics. By applying the RMS (the square root of the mean square) and STD (the standard deviation) complementary diagrams, it was found that the PA UV3 sample appears as a very unique sample with only one large affiliation (the dark highlighted value in Figure 10a) in the third group. This is a sample with a very high mean velocity (Figure 10b) and, in contrast, a small anisotropy (low STD value—Figure 10c). PD sample SAL1 has a similar small anisotropy. However, it does not have such high speeds and therefore represents a specific type in itself, defining another cluster, number 10. The sample MA HD10.3, which has an exceptional anisotropy (see the maximum measured standard deviation), defines cluster 1, to which samples such as PD LU5.3, PA SAL1, and several others with less similarity are assigned. Based on the above, several samples can be identified that are representative of “their” characteristic group. These are as follows: MA HD 10.3, PA LS 10.2, PA UV 3, MA UV 2, PA LU 5.1, PA LU 10.1, MA SAL 2, and MA LU 5.3. Cluster No. 7 is interesting in this respect, where two very similar velocity profiles are obtained from different wood species (Maple PA NC 10.1 and Spruce MD LU 15.2).

## 4. Conclusions

The use of non-destructive control to determine the sound propagation speeds in different stages of the finishing of musical instruments can be a modern and precise method of optimizing the finishing technology to obtain the desired acoustic features, depending on the quality of the wood, the type of varnish and the treatments applied. The results of this study help instrument manufacturers to integrate new technologies with the art of luthiers and also apply innovative solutions for treating resonance wood, which they can later verify using the ultrasound method. Based on the obtained experimental data, the following conclusions were drawn:

1. The treatment applied to the surface of the wooden resonance plates influences the sound propagation speed both in the longitudinal and radial directions. The acoustic behavior of the wood depends both on the type of treatment and the anatomical characteristics of the wood, with differences in propagation speeds being recorded between samples of the same woody species but with different anatomical structures.
2. In spruce wood samples, the film of alcohol varnish reduces the speed of propagation of sounds by approximately 42–58% in the longitudinal direction, and in the radial direction, the oil-based varnish reduces the speed of propagation by 60%.
3. In the maple wood samples, the anisotropy ratio between the longitudinal and radial directions decreases with the increase in the thickness of the varnish film.

## Figures and Tables

**Table 1:** Physical properties: wood species (WS), density (ρ), equilibrium moisture content (EMC), and type of treatment (T).

Samples Code	WS	ρ (kgm^−3^)	EMC (%)	AML (g/m^2^)	T
MDHD15.1	spruce	391.01 (33.42)	6.4 (0.4)	0.000	Untreated
MAHD10.3	spruce	470.83 (18.68)	7.9 (0.5)	0.000	
PDNC15.1	maple	605.72 (17.54)	6.1 (0.3)	0.000	
PANC10.1	maple	656.90 (15.82)	7.3 (0.5)	0.000	
MDUV2	spruce	421.50 (27.92)	6.8 (0.5)	−10.318	UV exposure 300 h
MAUV2	spruce	473.45 (18.32)	6.2 (0.4)	−13.979	
PDUV3	maple	565.40 (17.62)	6.2 (0.2)	−12.432	
PAUV3	maple	640.36 (11.75)	6.4 (0.7)	−17.813	
MDSAL2	spruce	429.33 (10.19)	8.3 (1.3)	16.047	Salt fog exposure NaCl 3.5% 72 h
MASAL2	spruce	491.16 (13.81)	8.8 (1.4)	21.563	
PDSAL1	maple	679.17 (35.24)	9.1 (1.2)	27.135	
PASAL1	maple	669.77 (24.71)	8.5 (1.8)	30.146	
MALU5.3	spruce	399.31 (45.60)	7.4 (0.9)	11.755	5 layers of oil-based varnish
MDLU5.2	spruce	407.85 (37.50)	7.8 (0.7)	9.807	
PALU5.1	maple	662.57 (33.14)	8.4 (0.6)	8.375	
PDLU5.3	maple	652.23 (19.20)	7.4 (0.5)	6.938	
MALU10.2	spruce	556.98 (29.40)	7.8 (0.3)	15.969	10 layers of oil-based varnish
MDLU10.2	spruce	496.42 (32.12)	7.3 (0.7)	17.526	
PALU10.1	maple	705.36 (41.24)	8.7 (0.3)	14.708	
PDLU10.1	maple	611.77 (32.15)	8.2 (0.4)	14.380	
MALU15.2	spruce	522.55 (21.35)	8.0 (0.5)	22.979	15 layers of oil-based varnish
MDLU15.2	spruce	511.99 (22.31)	7.9 (0.4)	23.599	
PALU15.1	maple	744.69 (34.32)	8.3 (0.3)	23.443	
PDLU15.1	maple	694.91 (21.12)	7.8 (0.6)	24.229	
MALS5.2	spruce	430.08 (36.23)	7.2 (0.4)	2.865	5 layers of spirit varnish
PALS5.2	maple	657.79 (15.43)	7.3 (0.2)	11.453	
MALS10.2	spruce	417.60 (18.45)	7.5 (0.2)	7.042	10 layers of spirit varnish
PALS10.2	maple	672.47 (21.34)	7.1 (0.3)	12.896	
MALS15.2	spruce	491.42 (35.82)	7.6 (0.5)	13.234	15 layers of spirit varnish
PALS15.2	maple	621.58 (28.12)	7.7 (0.2)	16.651	

## Data Availability

Data are contained within the article.
